# High Plasma Docosahexaenoic Acid Associated to Better Prognoses of Patients with Acute Decompensated Heart Failure with Preserved Ejection Fraction

**DOI:** 10.3390/nu13020371

**Published:** 2021-01-26

**Authors:** Naoaki Matsuo, Toru Miyoshi, Atsushi Takaishi, Takao Kishinoue, Kentaro Yasuhara, Masafumi Tanimoto, Yukari Nakano, Nobuhiko Onishi, Masayuki Ueeda, Hiroshi Ito

**Affiliations:** 1 Department of Cardiovascular Medicine, Okayama University Graduate School of Medicine, Dentistry and Pharmaceutical Sciences, Okayama 700-8558, Japan; naoaki.matsuo.1985@gmail.com (N.M.); itomd@md.okayama-u.ac.jp (H.I.); 2 Department of Cardiovascular Medicine, Mitoyo General Hospital, Kagawa 769-1601, Japan; takaishi1013@ybb.ne.jp (A.T.); takao.nakayama.0922@gmail.com (T.K.); ilovesukuramu@yahoo.co.jp (K.Y.); kpggp925@gmail.com (M.T.); nobuohnishi@mitoyo-hosp.jp (N.O.); 3 Nakano Cardiovascular Clinic, Kagawa 762-0012, Japan; nakanocc.20200129@gmail.com; 4 Ueeda Cardiovascular Clinic, Kagawa 769-1504, Japan; ueedacvc@gmail.com

**Keywords:** heart failure with preserved ejection fraction, docosahexaenoic acid, geriatric nutritional risk index

## Abstract

The clinical relevance of polyunsaturated fatty acids (PUFAs) in heart failure remains unclear. The aim of this study was to investigate the association between PUFA levels and the prognosis of patients with heart failure with preserved ejection fraction (HFpEF). This retrospective study included 140 hospitalized patients with acute decompensated HFpEF (median age 84.0 years, 42.9% men). The patients’ nutritional status was assessed, using the geriatric nutritional risk index (GNRI), and their plasma levels of eicosapentaenoic acid (EPA), docosahexaenoic acid (DHA), arachidonic acid (AA), and dihomo-gamma-linolenic acid (DGLA) were measured before discharge. The primary outcome was all-cause mortality. During a median follow-up of 23.3 months, the primary outcome occurred in 37 patients (26.4%). A Kaplan–Meier analysis showed that lower DHA and DGLA levels, but not EPA or AA levels, were significantly associated with an increase in all-cause death (log-rank; *p* < 0.001 and *p* = 0.040, respectively). A multivariate Cox regression analysis also revealed that DHA levels were significantly associated with the incidence of all-cause death (HR: 0.16, 95% CI: 0.06–0.44, *p* = 0.001), independent of the GNRI. Our results suggest that low plasma DHA levels may be a useful predictor of all-cause mortality and potential therapeutic target in patients with acute decompensated HFpEF.

## 1. Introduction

Heart failure (HF) is a common and growing public health problem with an estimated prevalence of over 37.7 million cases worldwide [1]. Despite recent developments of HF treatments, including pharmacological and device therapy, HF still results in high mortality and re-hospitalization rates [2]. HF clinically manifests in two modes, which are defined by ventricular function: HF with reduced ejection fraction (HFrEF) and HF with preserved ejection fraction (HFpEF) [3]. Unfortunately, standard pharmacological therapies for HFrEF such as angiotensin-converting enzyme inhibitors and β-blockers show a lack of efficacy in the treatment of HFpEF [4]. Patients with HFpEF are more likely to be older, female, and have hypertension, renal disease, atrial fibrillation, and malnutrition [5]. Malnutrition, in particular, is a common problem in elderly patients with HFpEF and is a known risk factor for a poor prognosis [6].

Polyunsaturated fatty acids (PUFAs) play structural and functional roles as membrane components and precursors of physiologically active substances involved in inflammation [7]. Fish oils, sunflower, safflower, and corn oils are rich in omega-3 PUFAs, while meat from farm animals are rich in omega-6 PUFAs [8]. Omega-3 PUFAs, such as eicosapentaenoic acid (EPA) and docosahexaenoic acid (DHA), and oemga-6 PUFAs, such as arachidonic acid (AA) and dihomo-gamma-linolenic acid (DGLA), have been shown to have opposite effect [9]. It has been reported that AA-derived metabolites are pro-inflammatory, while EPA- and DHA-derived metabolites are pro-resolution/anti-inflammatory [10,11,12]. Some metabolites have been reported to play a critical role in the development of cardiac hypertrophy and heart failure by regulating inflammatory reactions [12,13,14]. However, omega-7 and omega-9 monounsaturated fatty acids, such as palmitoleic acid and oleic acid, are components of complex lipids, such as sphingosines and phospholipids, and could interfere with cellular injury [15,16,17].

Several clinical trials and meta-analysis have demonstrated that omega-3 PUFAs are beneficial for patients with cardiovascular events [18,19,20]. Regarding the association between omega-3 PUFAs and heart failure, a meta-analysis of seven prospective studies with 176,441 subjects and 5480 cases of HF found a lower risk of HF in patients that took high amounts of marine omega-3 PUFAs [21]. Another study including 6562 patients, in over 13 years, found that plasma EPA levels were significantly lower in HF patients, compared to HF-free patients [22]. Small-scale clinical trials have indicated that omega-3 PUFAs may improve the outcomes of patients with HF [23,24,25,26]. However, recent large-scale randomized controlled studies investigating cardiovascular benefit of omega-3 supplementation showed conflicting findings [27,28].

The aim of this study was to investigate the role of PUFAs in the prognosis of patients with acute decompensated HFpEF. In addition, the impact of the patients’ nutritional status on the association between PUFAs and their prognosis was evaluated.

## 2. Materials and Methods

### 2.1. Study Design and Participants

**Figure 1:**
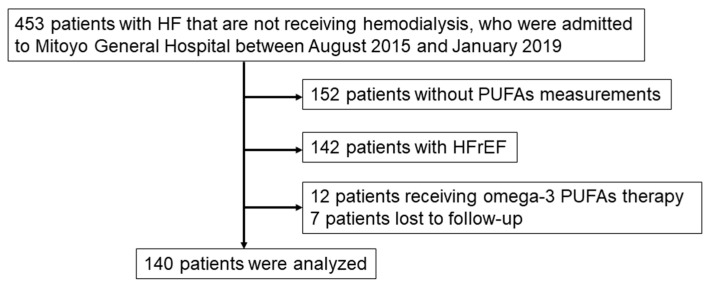
Flowchart of study population. Heart failure (HF) was defined based on the Framingham criteria. Heart failure with reduced ejection fraction (HFrEF); Heart failure with preserved ejection fraction (HFpEF) was defined as HF with a left ventricular ejection fraction ≥50%. PUFAs, polyunsatu-rated fatty acids.

This study was a retrospective single-center cohort study. The study protocol was approved by the Institutional Review Board of Mitoyo General Hospital (19CR01-122) and conducted in accordance with the principles of the Declaration of Helsinki. The requirement for informed consent was waived because of the low-risk nature of the study and inability to obtain consent directly from all the study subjects. Instead, we announced this study protocol extensively at Mitoyo General Hospital and on the hospital website (http://mitoyo-hosp.jp) and provided patients with the opportunity to withdraw from the study. We initially enrolled 301 consecutive patients with acute decompensated HFpEF that were not receiving hemodialysis and who were admitted to Mitoyo General Hospital between August 2015 and January 2019. Acute decompensated HF was diagnosed based on the Framingham’s criteria. [29]. A diagnosis of HF was made if a patient had at least two major criteria or one major criterion and two minor criteria. The major criteria are acute pulmonary edema, cardiomegaly, hepatojugular reflex, distended neck veins, paroxysmal nocturnal dyspnea, pulmonary rales, and third heart sound. The minor criteria are ankle edema, dyspnea on exertion, hepatomegaly, nocturnal cough, pleural effusion, and tachycardia [29]. HFpEF was defined as HF with a left ventricular ejection fraction ≥50%. Patients with HFrEF and those receiving omega-3 PUFA therapy were excluded. Figure 1 shows the flow diagram of this study. Follow-ups were performed by referring to patient electronic medical records, direct contact with the patients’ physicians in the outpatient clinic, and telephone interviews with patients or family members. A total of 140 patients were ultimately included in the final analysis.

### 2.2. Blood Sampling and Equations

Whole blood samples were collected within 24 h of admission. Approximately 20 mL of blood was collected by venipuncture and separated into tubes containing clot activator, gel serum separator, ethylenediaminetetraacetic acid dipotassium, and heparin sodium. Plasma levels of EPA, DHA, AA, and DGLA were measured by using gas chromatography (SRL Inc., Tokyo, Japan) [30]. Routine laboratory tests were performed, using an automated analyzer, at Mitoyo General Hospital. The estimated glomerular filtration rate (eGFR) was calculated based on the Japanese equation that uses serum creatinine level, age, and sex as follows: eGFR (mL/min/1.73 m^2^) = 194 × serum creatinine^−1.094^ × age^−0.287^ (for females = ×0.739) [31]. The geriatric nutritional risk index (GNRI) was calculated as follows, using the serum albumin level, body weight, and height obtained on admission: GNRI = 14.89 × serum albumin (g/dL) + 41.7 × (actual body weight/ideal body weight). GNRI is a nutrition-related risk index that makes it possible to classify patients according to a risk of morbidity and mortality, and the GNRI ≥98 means no nutritional-related risk [32]. The ideal body weight in the present study was calculated by using a body mass index of 22 kg/m^2^.

### 2.3. Assessment of Additional Risk Factors

Hypertension was defined as having a seated blood pressure >140/90 mmHg or undergoing current treatment with antihypertensive medications. Diabetes mellitus was defined as having a previous diagnosis of diabetes mellitus in the medical records, a hemoglobin A1C (national glycohemoglobin standardization program calculation) level ≥6.5%, or receiving treatment with oral antidiabetic agents or insulin. Dyslipidemia was defined as one or more of the following characteristics: ≥150 mg/dL serum triglyceride, <40 mg/dL high-density lipoprotein cholesterol (HDL-cholesterol), ≥140 mg/dL low-density lipoprotein cholesterol (LDL-cholesterol), or current treatment with a lipid-lowering drug. Smoking status was defined as “currently smoking”. 

### 2.4. Study Outcomes

The primary endpoint was all-cause mortality. Furthermore, as an ad hoc analysis, patients were divided into four groups, based on the median DHA level and median GNRI, so that the association between the primary endpoint and each group could be evaluated. The secondary endpoints were cardiac death and re-hospitalization for HF.

### 2.5. Statistical Analyses

The results are presented as the mean ± standard deviation when they are normally distributed, and as the median and interquartile range (IQR) when they are non-normally distributed. The normality of distribution was determined by the Kolmogorov–Smirnov test. Differences between the groups were analyzed by using the unpaired Student’s t-test or Mann–Whitney U test for continuous variables, and the chi-squared test or Fisher’s exact test for dichotomous variables, as appropriate. For the survival analyses, Kaplan–Meier survival plots were constructed by dividing the patients’ PUFA levels on admission into two groups, according to the median values, and log-rank testing was performed to study the influence of PUFA levels on primary and secondary endpoints. To evaluate the influence of PUFA levels on the primary endpoint, Cox proportional-hazards regression models were used to estimate the hazard ratio (HR) and 95% confidence interval (CI). To avoid overfitting, variables that were included in the principal multivariate models were adjusted for age, sex, hypertension, dyslipidemia, diabetes mellitus, and GNRI. All the tests were two-tailed, and a value of *p* < 0.05 was considered statistically significant. All the analyses were performed by using IBM SPSS statistics version 24.0 (IBM Corp., Armonk, NY, USA).

## 3. Results

### 3.1. Baseline Characteristics

Table 1 shows the baseline characteristics of the patients in this study and a comparison of those characteristics between the patients with and without primary endpoints. The median age of all the patients was 84.0 years, 42.9% were male, and 56.4% had atrial fibrillation. The prevalence of hypertension and diabetes mellitus within the group of patients was 90.0% and 22.9%, respectively. 

During the median follow-up of 23.3 months, 37 (26.4%) of the patients exhibited the primary endpoint. Patients experiencing the primary endpoint were older; had lower BMI and GNRI values; had a lower prevalence of hypertension and dyslipidemia; had lower statin use; and had lower hemoglobin, albumin, HDL-cholesterol, and LDL-cholesterol levels than those who did not experience the primary endpoint. No significant differences in the prevalence of atrial fibrillation, prior hospitalization for HF, or medication use, except for statins, were observed between the two groups. The median levels of EPA, DHA, DGLA, and AA, as well as the ratio of EPA to AA (EPA/AA), DHA to AA (DHA/AA), and AA + DGLA to EPA + DHA (AA + DGLA/EPA + DHA), on admission were 46.6 μg/mL, 116.1 μg/mL, 23.6 μg/mL, 159.8 μg/mL, 0.26, 0.74, and 1.15, respectively. The levels of DHA, DGLA, and AA for the patients with adverse events were significantly lower than for those patients without adverse events. The levels of EPA, EPA/AA, DHA/AA, and AA+DGLA/EPA+DHA did not differ between the two groups.

### 3.2. Cumulative Event Rates Based on PUFA Levels

**Figure 2:**
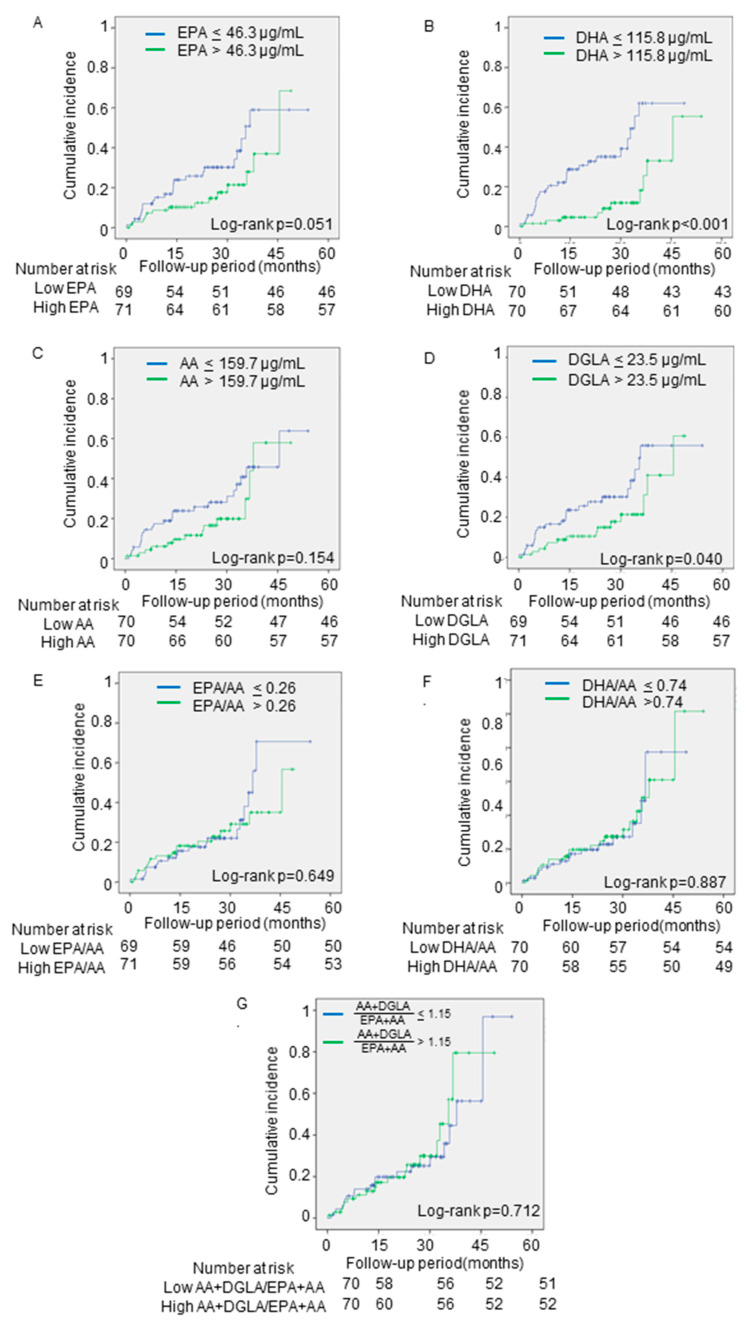
The associations between the primary outcomes and PUFA levels. The cumulative incidences of the primary endpoint (all-cause death) were estimated by using the Kaplan–Meier method. The patients were divided into two groups, based on the median levels of (**A**) EPA, (**B**) DHA, (**C**) AA, (**D**) DGLA, (**E**) EPA/AA, (**F**) DHA/AA, and (**G**) AA + DGLA/EPA + DHA. Log-rank testing was performed to study the influence of PUFA levels on primary endpoint. EPA, eicosapentaenoic acid; DHA, docosahexaenoic acid; DGLA, dihomo-gamma-linolenic acid; AA, arachidonic acid; EPA/AA, ratio of EPA to AA; DHA/AA, ratio of DHA to AA; AA + DGLA/EPA + DHA; ratio of AA + DGLA to EPA + DHA.

The Kaplan–Meier analyses showed that lower levels of DHA and DGLA on admission were significantly associated with the incidence of adverse events (log-rank; *p* < 0.001 and *p* = 0.040, respectively) (Figure 2B,D). However, the EPA and AA levels and the EPA/AA, DHA/AA, and AA + DGLA/ EPA + DHA were not associated (log-rank; *p* = 0.051, *p* = 0.154, *p* = 0.649, *p* = 0.887, *p* = 0.712, respectively) (Figure 2A,C,E–G).

### 3.3. Univariate and Multivariate Analyses of Parameters Contributing to the Primary and Secondary Endpoints

The univariate Cox regression analyses showed that age, body mass index, statin use, hemoglobin, albumin, LDL-cholesterol, GNRI, DGLA level, and DHA level were associated with the incidence of the primary endpoint (Table 2). The multivariate Cox regression analyses revealed that patients with high DHA levels was significantly associated with a low incidence of the primary endpoint after an adjustment for age, sex, hypertension, dyslipidemia, diabetes mellitus, and GNRI (HR: 0.16, 95% CI: 0.06–0.44, *p* = 0.001). However, the DGLA level was not significantly associated with the primary endpoint after an adjustment for confounding variables.

**Figure 3:**
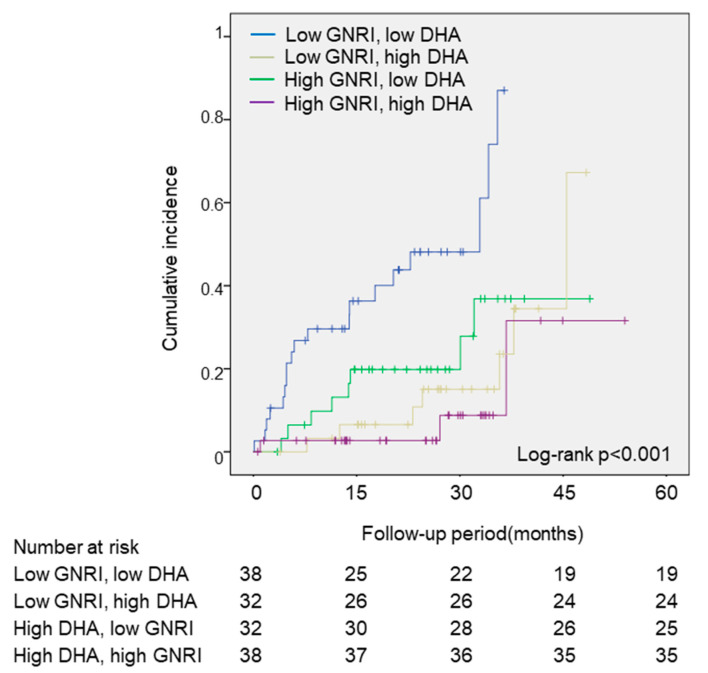
The associations between the primary outcomes and the DHA and GNRI values. The cumulative incidences of the primary endpoint (all-cause death) were estimated by using the Kaplan–Meier method. Log-rank testing was performed to study the influence of PUFA levels on primary endpoint. The patients were divided into four groups, based on the median DHA and GNRI values. DHA, docosahexaenoic acid; GNRI, geriatric nutritional risk index.

As an ad hoc analysis, the patients were divided into four groups, based on the median DHA and GNRI values. As shown in Figure 3, the low-GNRI and low-DHA groups showed the greatest incidence of the primary endpoint, compared to the other groups (log-rank; *p* < 0.001). In the multivariate Cox regression analyses, the low-GNRI and low-DHA groups had a significantly higher risk of the primary endpoint, compared with the high-GNRI and high-DHA groups, after an adjustment of age and sex (HR: 8.48, 95% CI: 2.47–29.07, *p* = 0.001) (Table 3).

**Figure 4:**
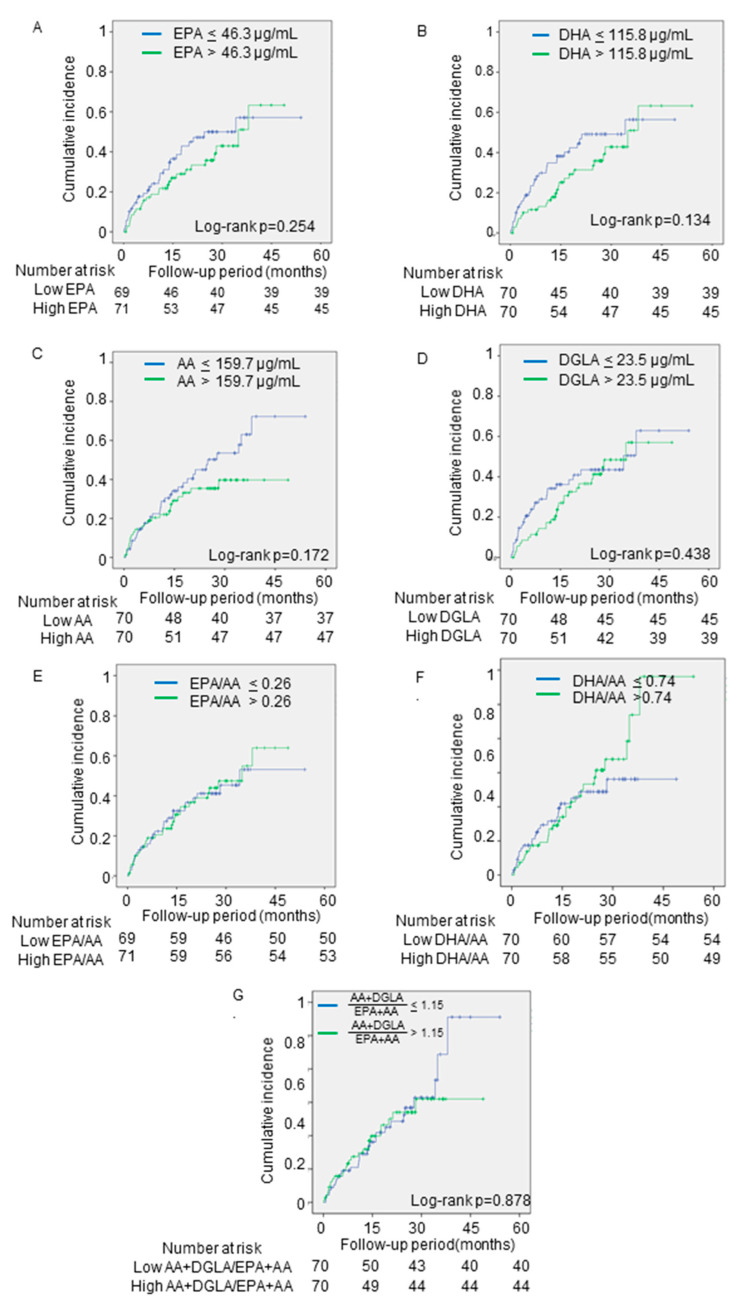
The associations between the secondary outcomes and PUFA levels. The cumulative incidences of the secondary endpoints (cardiac death and re-hospitalization for heart failure) were estimated by using the Kaplan–Meier method. Log-rank testing was performed to study the influence of PUFA levels on primary endpoint. The patients were divided into two groups, based on the median levels of (**A**) EPA, (**B**) DHA, (**C**) AA, (**D**) DGLA, and (**E**) EPA/AA, (**F**) DHA/AA, and (**G**) AA + DGLA/EPA + DHA. PUFA, polyunsaturated fatty acid; EPA, eicosapentaenoic acid; DHA, docosahexaenoic acid; AA, arachidonic acid; DGLA, dihomo-gamma-linolenic acid; EPA/AA, ratio of EPA to AA; DHA/AA, ratio of DHA to AA; AA + DGLA/EPA + DHA; ratio of AA + DGLA to EPA + DHA.

The secondary endpoints occurred in 63 patients (cardiac death (*n* = 15) and re-hospitalization for HF (*n* = 480)). As shown in Figure 4, none of the PUFA levels was associated with the secondary endpoints.

## 4. Discussion

The data from the present study showed that the acute decompensated HFpEF patients with lower plasma DHA levels had a significantly higher incidence of all-cause death, independent of GNRI. These findings suggest that plasma DHA levels are an important factor associated with prognosis, regardless of the nutritional status of patients with acute decompensated HFpEF. This suggests that measuring plasma DHA levels may be useful for the detection of high-risk patients hospitalized with HFpEF.

Several studies have shown the association between circulating concentrations of PUFAs and the incidence of HF. A previous cohort study, which included 2735 adults in the Cardiovascular Health Study from 1992 to 2006, reported that the total concentrations of omega-3 fatty acid were associated with the incidence of primary congestive HF [19]. A recent report from the Multi-Ethnic Study of Atherosclerosis (MESA) trial indicated that higher plasma EPA levels were significantly associated with a reduced risk of HF (for both reduced and preserved EF) [22]. In addition, regarding the association between PUFAs and the prognosis of patients with acute decompensated HF, a study showed that decreased plasma levels of DHA, DGLA, and AA were independently associated with long-term mortality in patients with acute decompensated HF [33]. Other studies have shown that lower omega-6 PUFAs levels were related to worse clinical outcomes in patients with acute decompensated HF [34,35]. However, most of the patients included in these studies had HFrEF. Thus, to the best of our knowledge, this is the first study to evaluate the correlation between PUFA levels and the prognosis of patients with HFpEF.

This study showed that lower DHA levels, but not EPA levels, were independently associated with all-cause mortality in patients with acute decompensated HFpEF. PUFAs play an important role in cellular membrane function [36]. While DHA is abundant in the cell membranes of cardiomyocytes [25], EPA is scarce. This difference may contribute to the distinct effects that DHA and EPA have on cardiac health. It should be noted, however, that while DHA can be obtained from the diet, it can also be synthesized from EPA [37]. In fact, the data from MESA suggested that EPA was more important than DHA for HF [19]. Therefore, any interpretation of the differences between the effects of DHA and EPA on the prognosis of HFpEF patients should be made with caution.

Although the present study showed a relationship between lower plasma DHA levels and a higher incidence of all-cause death, there was no significant association between DHA levels and composite events of cardiac death and re-hospitalization for HF. According to a Japanese cohort study called the Chronic Heart Failure Analysis and Registry in the Tohoku (CHART), the temporal trend in the mode of death in symptomatic HF has changed. As the prevalence of HFpEF in symptomatic HF increased from CHART-1 (2000–2005) to CHART-2 (2006–2010), the proportion of non-cardiac deaths increased from 23% in CHART-1 to 40% in CHART-2 [5]. In this study [5], those factors that were significantly associated with all-cause death were reported to be advanced age, low BMI, high systolic blood pressure, and absence of dyslipidemia. This is in line with our data shown in Table 1. Patients with HFpEF had more comorbidities than HFrEF patients, and noncardiac deaths occurred more frequently in HFpEF patients than in HFrEF patients [38].

Thus, the characteristics inherent to HFpEF patients specifically may be involved in the significant impact that DHA levels have on all-cause death, as opposed to cardiac death or re-hospitalization for HF.

Malnutrition is frequently observed and an important risk factor for poor outcomes in patients with HF. The GNRI is a simple and objective nutritional index, and a GNRI < 92 is generally used to evaluate the increased risk of morbidity and mortality in hospitalized elderly patients [21]. In our study, patients with the primary endpoint had an average GNRI of 90.8, suggesting a poor nutritional status. Although the patients with the primary endpoint also showed lower omega-3 PUFA levels, which were affected by oral intake, the Cox regression analyses revealed that the impact of the DHA levels on the patients’ prognoses was independent of the GNRI. Even in the patients with a poor nutritional status, lower DHA levels were shown to be an independent predictor of all-cause mortality in HFpEF patients.

Inflammation is a normal process that is part of the body’s defense and tissue-healing mechanism. However, excessive or unresolved inflammation can lead to uncontrolled tissue injury, and disease. Omega-6-derived metabolites, such as prostaglandins and leukotrienes, have pro-inflammatory effects, while omega-3-derived metabolites, such as resolvins and protectins, have anti-inflammatory and pro-resolving effects [10,11]. In this context, several clinical studies showed that the ratio of omega-3 to omega-6 PUFAs is a powerful predictor of heart disease [39,40,41]. Therefore, active screening of PUFAs would be beneficial in identifying patients at high risk of cardiovascular disease.

The GISSI-HF (Gruppo Italiano per lo Studio della Sopravvivenza nell’Infarto Miocardico Heart Failure) trial was a large-scale, placebo-controlled, randomized study that showed that 1 g daily of omega-3 fatty acid administration reduced the risk of all-cause death by 9% and the risk of hospitalization due to cardiovascular reasons by 8% in patients with chronic heart failure [42]. Other clinical trials have indicated that omega-3 fatty acids might improve outcomes in patients with HF [23,24,25,26]. In addition, animal studies have shown that omega-3 fatty acids, including EPA and DHA, at supraphysiological levels, preserve left ventricular function and prevent interstitial fibrosis in a mouse model of pressure overload-induced HF [39,43,44]. Despite these potential benefits, the use of omega-3 fatty acids in patients with HF remains controversial. Future large-scale randomized clinical trials to investigate the benefit of high dosages of omega-3 fatty acids, on top of the guideline-directed medical therapy for patients with documented overt HF, will be needed.

This study had several limitations. First, the study was conducted in a single center, the sample size was small, and the follow-up period was short. Therefore, it may be difficult to generalize these results. Second, the PUFAs were not measured in the cell membrane. PUFAs in the cell membrane have been reported to be direct precursors of pro- and anti-inflammatory eicosanoids. However, it has also been reported that cell-membrane PUFAs are significantly correlated with serum PUFAs in the Japanese population [30]. Third, food intake is associated with blood levels of PUFAs; however, measurement of dietary intake by using a frequency food questionnaire was not performed in this study. Moreover, the multivariate cox regression model included a limited number of variates to avoid statistical overfitting, because of the small number of the primary outcome. Therefore, large-scale studies will be needed to confirm our findings. Finally, the study was an observational study, so the causal relationship between DHA levels and prognosis is uncertain.

## 5. Conclusions

Lower levels of DHA are significantly associated with an increase in all-cause death in patients with acute decompensated HFpEF, independent of nutritional status. Measurement of plasma DHA levels may be useful in identifying high-risk patients with HFpEF, and supplementation with DHA may be a potential therapeutic target in these patients.

## Figures and Tables

**Table 1:** Baseline characteristics according to the presence or absence of the primary endpoint.

		Primary Endpoint		
Variables	All (*n* = 140)	Absent (*n* = 103)	Present (*n* = 37)	*p*
Men	60 (42.9)	40 (38.8)	20 (54.1)	0.110
Age, years	84.0 (77.0, 88.0)	82.0 (76.0, 88.0)	86.0 (83.0, 89.0)	0.018
Body mass index, kg/m^2^	23.3 (20.4, 26.6)	22.4 (20.8, 27.5)	22.1 (19.9, 24.1)	0.008
Hypertension	126 (90.0)	96 (93.2)	30 (81.1)	0.035
Diabetes Mellitus	32 (22.9)	22 (21.4)	10 (27.0)	0.485
Dyslipidemia	45 (32.1)	38 (36.9)	7 (18.9)	0.045
Current smoker	49 (35.0)	33 (32.0)	16 (43.2)	0.223
Prior hospitalization for heart failure	19 (13.6)	13 (12.6)	6 (16.2)	0.587
Ischemic heart disease	18 (12.9)	15 (14.6)	3 (8.1)	0.318
Atrial fibrillation	79 (56.4)	59 (57.3)	20 (54.1)	0.736
Prior PCI	14 (10.0)	12 (11.7)	2 (5.4)	0.281
Prior CABG	5 (3.6)	5 (4.9)	0 (0)	0.175
Valve repair/placement	14 (10.0)	12 (11.7)	2 (5.4)	0.281
Pacemaker implantation	14 (10.0)	7 (6.8)	7 (18.9)	0.035
Medications				
ACEIs/ARBs	55 (39.3)	43 (41.7)	12 (32.4)	0.323
β-blockers	48 (34.3)	39 (37.9)	9 (24.3)	0.139
CCBs	72 (51.4)	55 (53.4)	17 (45.9)	0.440
Loop diuretics	79 (56.4)	54 (52.4)	25 (67.6)	0.113
MRAs	26 (18.6)	16 (15.5)	10 (27.0)	0.125
Antiplatelets	31 (22.1)	27 (26.2)	4 (10.8)	0.053
Oral antidiabetic agents	22 (15.7)	17 (16.5)	5 (13.5)	0.671
Statins	35 (25.0)	31 (30.1)	4 (10.8)	0.020
Anticoagulants	22 (15.7)	46 (44.7)	17 (45.9)	0.894
Laboratory findings				
Hemoglobin (g/dL)	11.0 ± 2.09	11.2 ± 2.10	10.3 ± 1.94	0.017
Creatinine (mg/dL)	1.08 (0.82, 1.62)	1.03 (0.83, 1.56)	1.27 (0.79, 1.75)	0.364
eGFR (ml/min/1.73 m^2^)	42.2 (29.0, 56.0)	43.0 (29.0, 55.0)	41.0 (26.2, 59.6)	0.530
Albumin (g/dL)	3.6 (3.2, 3.9)	3.6 (3.4, 3.9)	3.4 (2.9, 3.7)	0.001
hsCRP (mg/dL)	0.43 (0.17, 1.45)	0.36 (0.15, 1.08)	0.86 (0.24, 1.59)	0.581
BNP (pg/mL)	453.0 (260.4, 699.0)	464.0 (246.8, 739.4)	411.0 (269.9, 659.5)	0.107
Troponin I (pg/mL)	32.7 (15.2, 84.7)	33.0 (14.3, 84.7)	28.9 (16.3, 70.8)	0.520
Hemoglobin A1C (%)	5.9 (5.6, 6.5)	5.9 (5.6, 6.5)	5.9 (5.6, 6.6)	0.871
Triglycerides (mg/dL)	76 (57, 98)	78 (62, 98)	67 (50, 94)	0.110
HDL-C (mg/dL)	46 ± 14.4	48 ± 13.8	41 ± 15.5	0.047
LDL-C (mg/dL)	97 ± 36.7	102 ± 37.1	81 ± 30.8	0.014
EPA (μg/mL)	46.6 (30.7, 64.2)	48.0 (31.9, 67.8)	39.6 (28.0, 55.0)	0.076
DHA (μg/mL)	116.1 (96.7, 144.9)	126.7 (99.1, 149.8)	102.8 (94.3, 119.5)	0.009
AA (μg/mL)	159.8 (133.8, 194.5)	167.1 (144.2, 195.0)	139.1 (110.5, 180.7)	0.001
DGLA (μg/mL)	23.6 (19.6, 30.5)	24.2 (20.1, 32.5)	22.2 (18.4, 27.1)	0.019
EPA/AA	0.26 (0.20, 0.40)	0.27 (0.20, 0.40)	0.26 (0.21, 0.39)	0.812
DHA/AA	0.74 (0.62, 0.89)	0.74 (0.60, 0.90)	0.78 (0.63, 0.88)	0.287
AA + DGLA/EPA + DHA	1.15 (0.90, 1.38)	1.15 (0.90, 1.42)	1.14 (0.95, 1.32)	0.481
GNRI	97.7 ± 12.03	99.9 ± 11.59	90.8 ± 10.84	< 0.001

Categorical variables are presented as number of patients (%). Continuous variables are presented as the mean ± standard deviation or median (interquartile range). PCI, percutaneous coronary intervention; CABG, coronary artery bypass grafting; ACEs, angiotensin-converting enzyme inhibitors; ARBs, angiotensin II receptor blockers; CCBs, calcium channel blockers; MRAs, mineralocorticoid receptor antagonists; eGFR, estimated glomerular filtration rate; hsCRP, high-sensitivity C-reactive protein; BNP, brain natriuretic peptide; HDL-C, high-density lipoprotein cholesterol; LDL-C, low-density lipoprotein cholesterol; EPA, eicosapentaenoic acid; DHA, docosahexaenoic acid; DGLA, dihomo-gamma-linolenic acid; AA, arachidonic acid; DHA/AA, ratio of DHA to AA; AA+DGLA/EPA+DHA, ratio of AA+DGLA to EPA+DHA; GNRI, geriatric nutritional risk index.

**Table 2:** The association between PUFAs and the primary endpoint analyzed with Cox proportional hazards models.

		Univariate		Multivariate-1			Multivariate-2		
	HR	95% CI	*p*	HR	95% CI	*p*	HR	95% CI	*p*
Age, per 1 year	1.06	1.01–1.11	0.009	1.05	0.99–1.10	1.102	1.06	1.00–1.13	0.042
Male	1.76	0.92–3.37	0.087	1.76	0.81–3.83	0.155	1.98	0.91–4.29	0.083
Body mass index, per 1.0 kg/m^2^	0.90	0.82–0.98	0.017	-	-	-	-	-	-
Hypertension	0.52	0.26–1.06	0.071	0.65	0.26–1.59	0.342	1.13	0.45–2.82	0.793
Dyslipidemia	0.45	0.20–1.03	0.060	0.72	0.25–2.12	0.549	0.66	0.23–1.91	0.441
Diabetes mellitus	1.54	0.74–3.21	0.249	1.99	0.87–4.58	0.104	1.86	0.78–4.46	0.164
Statin use	0.34	0.12–0.95	0.040	-	-	-	-	-	-
Hemoglobin, per 1.0 mg/dL	0.82	0.70–0.96	0.013	-	-	-	-	-	-
Albumin, per 1.0 g/dL	0.34	0.18–0.63	0.001	-	-	-	-	-	-
HDL-C, per 1 mg/dL	0.96	0.93–1.00	0.054	-	-	-	-	-	-
LDL-C, per 1 mg/dL	0.98	0.96–0.99	0.004	-	-	-	-	-	-
GNRI, per 1 index	0.94	0.91–0.97	<0.001	0.95	0.91–0.99	0.010	0.95	0.91–0.98	0.002
High DGLA	0.50	0.26–0.98	0.044	1.02	0.47–2.19	0.969	-	-	-
High AA	0.61	0.31–1.21	0.158	-	-	-	-	-	-
High EPA	0.52	0.27–1.01	0.055	-	-	-	-	-	-
High DHA	0.25	0.12–0.53	<0.001	-	-	-	0.16	0.06–0.44	<0.001
High EPA/AA	0.86	0.45–1.65	0.650	-	-	-		-	
High DHA/AA	1.05	0.54–2.02	0.887	-	-	-		-	
High AA + DGLA/ EPA + DHA	1.13	0.59–2.17	0.712	-	-	-		-	

The multivariate model-1 and model-2 were adjusted for age, sex, hypertension, dyslipidemia, diabetes mellitus, and GNRI. HR, hazard ratio; CI, confidence interval; GNRI, geriatric nutritional risk index; EPA, eicosapentaenoic acid; DHA, docosahexaenoic acid; DGLA, dihomo-gamma-linolenic acid; AA, arachidonic acid. EPA/AA, ratio of EPA to AA; DHA/AA, ratio of DHA to AA; AA + DGLA/EPA + DHA; ratio of AA + DGLA to EPA + DHA.

**Table 3:** The association between the DHA and GNRI values and primary endpoints analyzed with Cox proportional hazards models.

	Multivariate Analysis		
Variables	HR	95% CI	*P*
Age, per 1-year	1.07	1.02–1.13	0.006
Male	1.67	0.87–3.22	0.123
High DHA and high GNRI	Reference		
High DHA and low GNRI	1.14	0.28–4.64	0.858
Low DHA and high GNRI	3.03	0.80–11.48	0.104
Low DHA and low GNRI	8.48	2.47–29.07	0.001

Multivariate analysis was adjusted by age, sex, hemoglobin, and GNRI. HR, hazard ratio; CI, confidence interval; DHA, docosahexaenoic acid; GNRI, geriatric nutritional risk index.

## Data Availability

The data presented in this study are available on request from the corresponding author.
